# Jottings

**Published:** 1910-01-08

**Authors:** 


### Jottings.

The members of the ward of Queenhithe have appointed Mr. Edgar E. Bond (Queenhithe) to be a governor of St. Bartholomew's Hospital, in the place of Mr. C. A. Teuten, deceased.

The After-Care Association for Poor Persons Discharged Recovered from Asylums for the Insane appeals for funds to carry on and extend the work, the objects of which are to assist in various ways recovered cases on their discharge from asylums. Secretary, H. Thornhill Roxby, Church House, Westminster, S.W. Bankers, Union of London and Smiths, 117 Victoria Street, S.W.

The Metropolitan Hospital, Kingsland Road, was reopened by the Lord Mayor on December 20. Lord Howard de Walden presided.

A report of the Finance Committee of the Hospital Saturday Fund, which was presented on New Year's Day, gives the receipts and payments from January 12 to December 11 of 1909 compared with 1908. In these 11 months in 1908 the receipts amounted to £20,413 and the payments to £2,206, leaving a balance of £18,207, which, with a sum of £594 in hand from 1907, made a total credit balance of £18,801. The corresponding figure this year is £18,647. The decrease of £154 is almost exactly accounted for by the increase of payments to £2,360. The actual receipts for the 11 months were £20,442, about £28 more than in 1908; but, on the other hand, the credit balance from last year was less by that sum than it was 12 months ago. It is intimated to collectors and subscribers that the accounts for 1909 will be closed on Monday, January 10 next, at 6 p.m.

Melbourne Hospital Saturday and Sunday collections resulted in a total of £7,555 9s. 7d. in revenue.

The Essex Educational Committee has decided that there should be systematic medical inspection of scholars attending secondary schools in the county, and a special committee has been appointed to report on details.

